# Back Extensor Strength as a Potential Marker of Frailty Using Propensity Score Matching and Machine Learning

**DOI:** 10.3390/jcm12196156

**Published:** 2023-09-24

**Authors:** Taewook Kim, Gowun Kim, Hee-won Park, Eun Kyoung Kang, Sora Baek

**Affiliations:** 1 Department of Education & Human Resources Development, Seoul National University Hospital, Seoul 03080, Republic of Korea; ray0601@snu.ac.kr; 2 Department of Rehabilitation Medicine, Kangwon National University College of Medicine, Chuncheon-si 24341, Republic of Korea; gowun85@gmail.com (G.K.); hwp9980@gmail.com (H.-w.P.); 3 Department of Rehabilitation Medicine, Kangwon National University Hospital, Chuncheon-si 24289, Republic of Korea; 4 Center for Farmers’ Safety and Health, Kangwon National University Hospital, Chuncheon-si 24289, Republic of Korea; 5 Technological Laboratory, KakaoHealthcare Corp., Seongnam-si 13529, Republic of Korea; stewardofgod@gmail.com

**Keywords:** muscle strength, frailty, machine learning

## Abstract

This study assessed the potential of back extensor strength as an alternative marker of frailty. A total of 560 farmers were included. Computed tomography scans measured fat and muscle mass volumes at the mid-L4 vertebral level. Back extensor strength was measured in a seated posture. Multivariate linear regression was used to analyze the associations between back extensor strength and trunk muscle/fat compositions. The participants were divided into two groups based on back extensor strength. Propensity score matching, multivariate logistic regression, and Extreme Gradient Boosting (XGBoost) were employed to evaluate the relationship between Fried’s frailty criteria and back extensor strength. Back extensor strength exhibited positive associations with abdominal muscle volume (r = 1.12) as well as back muscle volume (r = 0.89) (*p* < 0.05). Back extensor strength was linked to more frail status, such as reduced grip strength, walking speed, and frequent self-reported exhaustion. Multivariate logistic regression indicated that back extensor strength was associated with higher frail status (OR = 0.990), and XGBoost analysis identified back extensor strength as the most important predictor (gain = 0.502) for frailty. The prediction models using grip strength produced similar results (OR = 0.869, gain = 0.482). These findings suggested the potential of back extensor strength as an alternative frailty marker.

## 1. Introduction

Frailty is a geriatric condition characterized by an increased vulnerability to external stressors [1]. It is associated with adverse health outcomes, including higher mortality rates, increased risk of falls, and chronic diseases [2]. In 2001, Fried proposed a frailty assessment using five physical components, including grip strength [3]. Subsequently, various frailty classifications have been developed and implemented in clinical and research settings [4,5], however, Fried’s frailty assessment remains widely used and recognized [6,7].

After Fried proposed grip strength as a frailty criterion, it has been shown to be an objective and reliable predictor of health outcomes [8,9]. However, grip strength is influenced by various factors beyond aging-related changes; lifestyle factors, such as nutrition and depression, have also been shown to impact grip strength [10,11]. Studies conducted in 11 European countries have revealed that even economic crises, such as a decrease in gross domestic product (GDP), can be associated with reduced grip strength [12]. Therefore, grip strength is not solely reflective of age-related decline but is influenced by a complex interplay of individual, societal, and economic factors. These findings highlight the multifactorial nature of grip strength and emphasize the need to consider a comprehensive range of determinants when interpreting its significance as a frailty marker.

As an alternative approach, researchers have studied muscle strength in different body parts beyond hand grip to capture a more comprehensive understanding of an individual’s physical function and overall frailty status [13]. Furthermore, the development of newly advanced dynamometers has enabled a wide range of muscle strength measurements, providing new possibilities for assessing muscle strength in multiple parts of the body [14,15].

This study focused on the back extensor muscles that play a crucial role in maintaining posture, stability, and overall trunk function [16]. Previous studies have shown that trunk muscle strength is associated with pulmonary dysfunction, a tendency to fall, dynamic balance, and lower extremity function, all of which can impact daily life [17,18,19,20]. Weakness or dysfunction in these muscles may contribute to balance issues, gait abnormalities, and increased vulnerability to falls, which are key components of frailty [21]. Hence, understanding the impact of back extensor strength on frailty could have significant clinical implications.

This study aimed to establish a correlation between frailty status and back extensor muscle strength. To assess the utility of incorporating back extensor muscle strength into frailty assessments, we analyzed both traditional logistic models and cutting-edge machine learning models to predict frailty using back extensor strength. We then compared these models with those using grip strength to predict frailty. Additionally, we examined the relationship between back extensor strength and trunk muscle/fat compositions to elucidate the rationale behind incorporating back extensor strength into frailty assessment. Through these analyses, our study seeks to enhance our understanding on the role played by back extensor muscle strength in frailty and its potential usefulness as a frailty marker.

## 2. Materials and Methods

### 2.1. Study Design

This cross-sectional study was conducted as an extension of the FARM (Farmers’ Cohort for Agricultural Work-Related Musculoskeletal Disorders) study, with the goal of evaluating musculoskeletal disorders among farmers in Gangwon Province, Republic of Korea. The baseline study took place between September 2013 and June 2014 [22], followed by the second wave survey of the cohort performed between October 2014 and March 2015 [23].

### 2.2. Ethical Considerations

This study protocol was reviewed and approved by the Institutional Review Board of Kangwon National University Hospital (approval no. 2016-03-008). Written informed consent was obtained from all participants.

### 2.3. Participants

**Figure 1:**
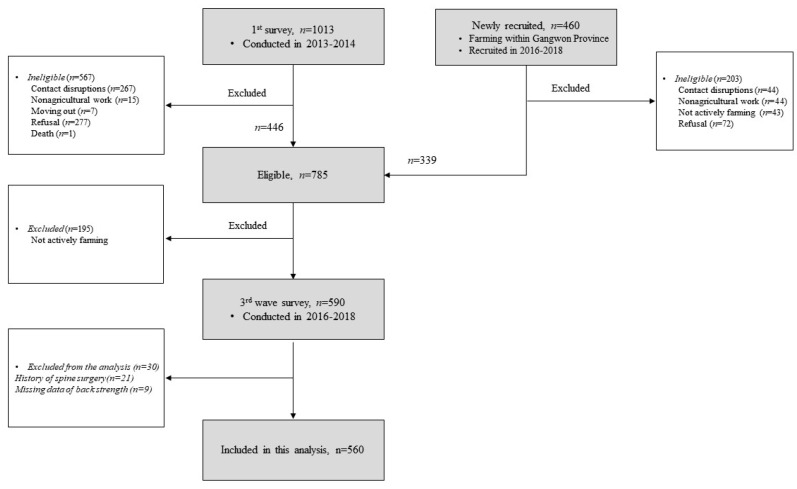
Flow chart for participant selection.

To select participants for the current study, we initially identified eligible individuals from the FARM cohort. Owing to various reasons such as changes in contact information, relocation, or deaths, some participants were ineligible. Consequently, 446 eligible participants were selected from the FARM cohort. We also recruited 339 new eligible individuals engaged in farming between 2016 and 2018. In consideration of the physically demanding nature and varied ergonomic tasks involved in farming [24,25] that contribute to specific changes in muscle volume, individuals who were not engaged in farming were excluded from the study. Among the initially identified 785 eligible participants, we excluded 195 non-active farmers, resulting in a total of 590 farmers who ultimately participated in the current assessment from 2016 to 2018. Of 590 individuals who participated in this study, we attempted to minimize the impact of external factors that could affect muscle volume by excluding 21 participants who had a history of spine surgery, a factor known to lead to paraspinal muscle atrophy [26]. Additionally, we excluded 9 participants owing to missing data on back extensor strength, resulting in a final analysis involving 560 participants (Figure 1). Sociodemographic, health characteristics, and agricultural work-related factors were assessed using a structured questionnaire and interview. In addition, laboratory examinations, including lumbar spinal radiography and serologic testing, were conducted.

### 2.4. Outcomes

#### 2.4.1. Frailty Score

Frailty was measured based on Fried’s five criteria. Each criterion had a value of 1 or 0: weight loss (weight loss in the past year ≥4.5 kg), self-reported exhaustion (at least 3 days a week), low physical activity (metabolic equivalent within the lowest 20%, adjusted for sex), slowness (usual 4-m gait speed within the lowest 20%, adjusted for sex and height), and weakness (grip strength within the lowest 20%, adjusted for body mass index [BMI] and sex). Based on the number of satisfied criteria, participants were divided into two groups: non-frail (frailty score of 0–2) and frail (frailty score of 3–5).

Exhaustion was defined based on the answer to the following question from the Center for Epidemiological Studies Depression Scale: “I felt all I did was an effort” for 3 or more days per week. Physical activity was assessed using the Korean version of the International Physical Activity Questionnaire [26]. To determine slowness, participants were divided into four groups based on sex and the median value of height, and the lowest 20% of the 4-m gait speed was calculated for each group and used as the cutoff value for slowness. Grip strength was measured three times on both hands, and the mean of these three values was calculated for each hand. The higher mean grip strength from both hands was selected for further analysis. To determine weakness in grip strength, participants were divided into four groups based on sex and the median BMI value, and the lowest 20% of the grip strength was calculated for each group and used as the cutoff value for weakness. 

#### 2.4.2. Back Extensor Strength

**Figure 2:**
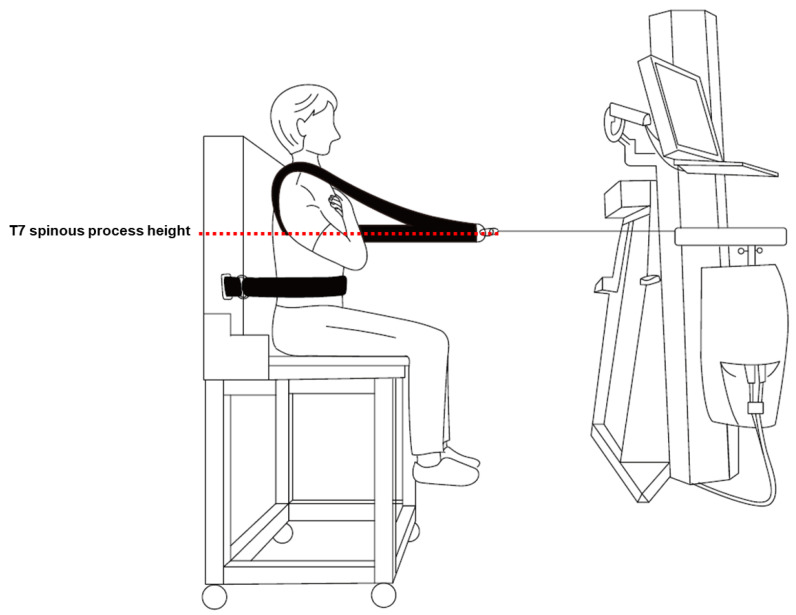
Descriptive figure for back extensor strength measurement.

The isometric strength of the back extensor was measured using the PrimusRS system (BTE Technologies Inc., Hanover, MD, USA) while participants were seated in a high chair to prevent their feet from touching the floor [27]. The height of the anchoring cable was modified to the height of the participants’ T7 spinous process, and a seatbelt was used to stabilize the participants’ thighs. Participants were asked to push back as far as possible, which took 3 s with isometric back extension. The cable anchored to the chest harness was pulled, and the isometric strength was measured (Figure 2). This process was repeated five times, and the mean of the three median values (excluding the maximum and minimum values) was used.

#### 2.4.3. Trunk Muscle/Fat Mass

The trunk muscle/fat mass measurement protocol in this study has been previously explained [28]. Ten consecutive computed tomography (CT) images at the mid-L4 vertebral level were acquired using a Philips MX 8000 IDT CT scanner (Philips Medical Systems, Cleveland, OH, USA), with exposure at 200 mAs, tube voltage set at 120 kV, and 1 mm slice thickness. Total muscle mass (TMM, cm^3^) and total fat mass (TFM, cm^3^) in the trunk region were obtained using image processing software (Extended Brilliance Workspace version 4.5.3; Philips Healthcare, Best, The Netherlands). Pre-defined radiation attenuation ranges were used to demarcate adipose (from −190 HU to −30 HU) and muscle (from −29 HU to +150 HU) tissues. TMM was subdivided into back muscle mass (BMM, cm^3^), psoas muscle mass (PMM, cm^3^), and abdominal muscle mass (AMM, cm^3^). BMM and PMM were derived manually from the TMM. AMM was obtained by subtracting the PMM and BMM from the TMM. The BMM consisted of the multifidus, iliocostalis lumborum, longissimus, and quadratus lumborum muscles. Visceral fat mass (VFM, cm^3^) was calculated by manually outlining the inner abdominal wall, and subcutaneous fat mass (SFM, cm^3^) was calculated by subtracting the VFM from the TFM. To reduce bias, one technician performed all scan and image processing procedures.

### 2.5. Statistical Analyses

All analyses were performed using R version 4.2.2. (The R Foundation, Vienna, Austria), Windows 10 [29]. Statistical significance was set at *p* < 0.05. Baseline characteristics of the study participants were presented and compared in two ways: between male and female and between those aged <65 years and age ≥65 years. Categorical variables are expressed as numbers and percentages and were compared using the chi-squared test. Continuous variables are expressed as means and standard deviations and were compared using the Student’s *t*-test. A linear regression analysis was performed to examine the relationship between trunk muscle/fat composition and back extensor strength. We analyzed the presence of multicollinearity using the variance inflation factor (VIF), with covariates having absolute values of VIF > 10 indicating multicollinearity [30].

To determine the relative importance of covariates in linear regression, relative weight analysis (RWA) was calculated [31]. The absolute value of the RWA was used to indicate the correlation between the covariate and back extensor strength, with positive and negative RWA values indicating a positive and negative relationship with back extensor strength, respectively.

#### 2.5.1. Propensity Score Matching 

The participants were divided into two groups based on their back extensor strength, with the lowest 20% strength considered as one group and the rest as the higher group. Because the result of the descriptive analysis, indicated a significant relationship between sex, age, frailty, and back extensor strength, we performed propensity score (PS) matching of age and sex. The purpose of PS matching was to reduce confounding effects by adjusting for confounding factors. PS is the conditional probability of an individual being in a certain group, based on covariates or predictor variables in the model [32]. Many researchers have used PS matching in medical and social science studies to reduce selection bias [33,34]. The nearest neighbor (NN) with a PS caliper, which imposed a tolerance level on the maximum PS distance, was used to match the two groups to reduce the risk of bad matches when the NN was located far away [35]. The matching ratio between the two groups was set as high as possible to increase the number of participants in the analysis. The standardized mean difference (SMD) of covariates was used to evaluate how well the two groups were paired, and an SMD < 0.1 was accepted as significantly reduced confounding bias [36].

#### 2.5.2. Multivariate Logistic Regression

To determine the potential of back extensor strength as a frailty parameter, we conducted a multivariate logistic regression analysis of frailty. We included age, sex, and BMI as covariates in frailty prediction, as these factors are commonly used covariates in strength assessments of frailty [37]. Odds ratios (OR) and 95% confidence intervals (CI) were calculated. Furthermore, to compare the results obtained with back extensor strength, we conducted prediction models in the same way using grip strength.

#### 2.5.3. Extreme Gradient Boosting

To demonstrate the possibility of using back extensor strength as a frailty parameter, we performed Extreme Gradient Boosting (XGBoost) [38]. XGBoost is a novel gradient-boosting algorithm that has been proven to enhance the computing power of classification and regression models. The XGBoost model for frailty prediction was performed in two steps: the grid search method, which was used to determine the optimal XGBoost hyperparameters, and the optimal XGBoost model, which was repeated 100 times to calculate the CI of the performance parameters.

First, we used the grid search method to optimize the XGBoost hyperparameters (maximum depth of a tree, learning rate, gamma, subsample ratio of the training instance, minimum sum of instance weight, and subsample ratio of columns). For the grid search, the maximum depth of a tree was set to 2, 4, 6, 8, 10, and 12; the learning rate was set to 0.001, 0.0025, 0.005, 0.01, 0.02, 0.04, 0.06, 0.08, 0.1, 0.2, and 0.5; gamma was set to 0, 0.1, 0.5, 1, 2, and 5; the subsample ratio of the training instance was set to 0.75 and 1; the minimum sum of instance weights was set to 1, 2, and 3; and the subsample ratio of columns was set to 0.75 and 1. Approximately 4752 combinations of possible hyperparameters were used in XGBoost, and the root mean square error (RMSE) of each model was compared.

To prevent overfitting, we used five-fold cross-validation. Our study population was split into five groups (“five folds”) equally. In the first iteration, the first fold was used as the test dataset, and the other four folds were used as the training dataset. Using a single combination of hyperparameters, the process was repeated until all folds were used as a test dataset (five iterations). The RMSE of XGBoost was calculated by averaging the results of the five iterations, and the RMSEs of all XGBoost models were compared. The minimum RMSE of XGBoost was obtained: learning rate was 0.2, maximum depth of a tree was 2, gamma was 2, subsample ratio of columns was 0.75, minimum sum of instance weight was 3, and subsample ratio of the training instance was 0.75.

The optimal hyperparameters were then used to perform XGBoost to evaluate frailty with back extensor strength. The optimal XGBoost model was trained to evaluate the group with frailty (frailty score ≥ 3). In this study, 70% of the participants were used as the training set, and 30% of the participants were used as the test set. The cutoff value for frailty prediction was selected based on Youden’s index [39]. The performance of the optimal XGBoost model was evaluated by calculating the area under the receiver operating characteristic curve [40], with an area under the curve (AUC) of 0.5 considered as null accuracy, and a higher AUC considered as better accuracy. Other classification evaluation metrics, such as accuracy, precision, recall, and f1 score, were also evaluated to assess the predictive power of back extensor strength in the XGBoost classification. 

The feature importance of each variable was estimated, and the model provided “gain” as the importance of the feature in the frailty prediction tree branches [41]. The gain for each feature was calculated by dividing the sum of the gains for the feature by the sum of the gains for all the features. Features with higher gains were considered more important in XGBoost model construction. Because XGBoost, similar to many other machine learning algorithms, employs random initialization during tree model construction, these random processes can result in relatively small variations in the model’s performance. As suggested by previous studies utilizing XGBoost in prediction models [34,42], we conducted analyses 100 times, allowing us to reduce these effects and provide more accurate estimates of 95% CIs.

To compare the results obtained with back extensor strength, we conducted prediction models in the same way using grip strength.

## 3. Results

### 3.1. Participant Characteristics

Of the 560 participants, 255 were male, and 305 were female (Table 1). The average age of the participants was 58.0 ± 7.0 years, with an average of 58.5 ± 7.0 years for males and 57.5 ± 6.9 years for females. The muscle/fat compositions, such as TMM, BMM, PMM, AMM, VFM, and SFM, differed between males and females. Female participants had a smaller waist circumference than males (*p* < 0.001). The CT results showed higher VFM in males (*p* < 0.001) and higher SFM in females (*p* < 0.001). Male participants had higher TMM, BMM, PMM, and AMM values (*p* < 0.001). In terms of frailty, males exhibited a faster walking speed (*p* < 0.05), higher grip strength, and higher back extensor strength (*p* < 0.001). There were no significant differences between sexes in terms of age, BMI, TFM, unintentional weight loss, self-reported exhaustion, physical activity, or Fried’s frailty score.

The participants were divided into two categories based on age to capture the impact of aging on trunk muscle/fat compositions (Table 2). There were 90 participants aged ≥65 years, and 470 participants aged <65 years. The 65-years-old cutoff in this study was based on the definition of an aging society in Korea [43]. The CT results showed higher TMM, PMM (*p* < 0.05), and BMM (*p* < 0.001) in participants aged <65 years. Furthermore, grip strength, back extensor strength (*p* < 0.05), and walking speed (*p* < 0.001) were higher in the <65 years group than in the ≥65-years group. The frailty score was higher in participants aged ≥65 years than in those aged <65 years (*p* < 0.001). Waist circumference, BMI, fat mass measurements, AMM, unintentional weight loss, self-reported exhaustion, and physical activity showed no significant differences between the age groups.

### 3.2. Linear Regression Analysis of Trunk Muscle/Fat Compositions and Back Extensor Strength

Table 3 shows the results of multivariate linear regression. Because age and sex had confounding effects (Table 1 and Table 2), these factors were adjusted for in the regression analysis. After the adjustment, a clear trend was observed (*p* < 0.05) in which higher back extensor strength was associated with increased AMM (r = 1.12) and BMM (r = 0.89). The PMM, VFM, and SFM were not significantly associated with back extensor strength. Each absolute value of the VIF was <10, indicating that there was no multicollinearity issue. A relative weight analysis showed that AMM (relative weight = 0.089) was the most significant predictor among the trunk muscle/fat components, and being female compared with male (relative weight = −0.118) was the most important predictor among all the parameters used.

### 3.3. PS Matching of the Group with the Lowest 20% Back Extensor Strength

The characteristics of the two groups were compared. One group had a lower back extensor strength of 20%, whereas the other group had a higher back extensor strength. Before PS matching, the age and sex covariates showed an SMD > 0.1 (Table 4). After PS matching (with a match ratio of 1:3 and caliper of 0.01), 108 participants from the lowest 20% group and 279 participants from the higher group remained with an SMD < 0.1. Differences between the two groups were assessed using the *t*-test for continuous variables and the chi-square test for categorical variables. Table 4 shows that grip strength, self-reported exhaustion, and walking speed significantly differed (*p* < 0.05) between the two groups, with the lowest 20% back extensor strength group showing greater frailty. 

### 3.4. Back Extensor Strength as a New Predictor of Frailty

The results of the multivariate logistic regression showed that higher back extensor strength was significantly associated with lower odds of frailty (OR, 0.990; 95% CI, 0.983–0.997; *p* < 0.05), whereas aging was associated with increased odds of frailty (OR, 1.088; 95% CI 1.025–1.160; *p* < 0.05). The analysis included sex and BMI as confounding factors; however, these factors were not significant predictors of frailty (Table 5). The multivariate logistic regression performed with grip strength showed similar results. Higher grip strength was significantly associated with lower odds of frailty (OR, 0.869; 95% CI, 0.805–0.933; *p* < 0.05) (Table 6).

**Figure 3:**
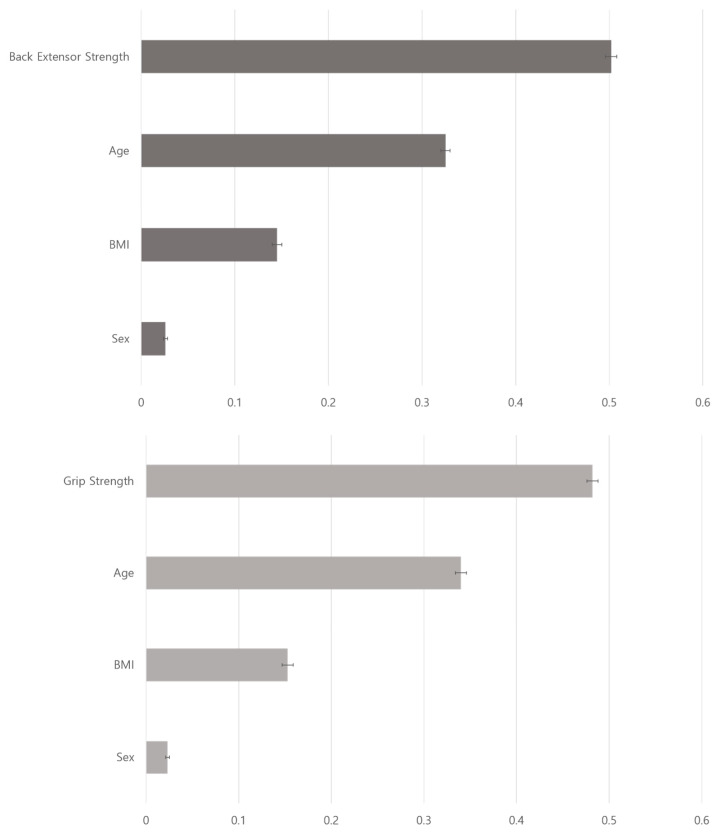
The feature importance ranking of XGBoost for frailty using back extensor strength (**upper**) and grip strength (**lower**). XGBoost, Extreme Gradient Boosting; BMI, body mass index; Sex, female.

The results of XGBoost for frailty prediction (frailty score ≥ 3 or not) showed that back extensor strength was the most important predictor of frailty (gain = 0.502 ± 0.006) and was more important than age (gain = 0.325 ± 0.005), BMI (gain = 0.145 ± 0.005), and sex (gain = 0.026 ± 0.002). The XGBoost model had an AUC of 0.579 ± 0.004, accuracy of 0.71 ± 0.05, precision of 0.10 ± 0.01, recall of 0.56 ± 0.04, and f1 score of 0.15 ± 0.01 (Figure 3 and Table 7). The XGBoost model performed using grip strength showed similar results; grip strength was the most important predictor of frailty (gain = 0.482 ± 0.007), and the model had an AUC of 0.676 ± 0.005, accuracy of 0.68 ± 0.02, precision of 0.09 ± 0.01, recall of 0.73 ± 0.02, and f1 score of 0.15 ± 0.01 (Figure 3 and Table 8).

## 4. Discussion

This study aimed to assess the potential of back extensor strength as an alternative marker of frailty, and the results revealed a significant association between lower back extensor strength and reduced muscle volume in both the abdominal and back muscles. Additionally, participants with lower back extensor strength exhibited higher levels of frailty according to Fried’s criteria, which was characterized by weaker grip strength, slower walking speed, and more frequent feelings of exhaustion. Both the multivariate logistic regression and the XGBoost model analyses consistently demonstrated that back extensor strength was a highly significant factor of frailty and not inferior to the prediction model using grip strength. Notably, the study revealed that the importance of back extensor strength in predicting frailty was greater than that of age alone, suggesting that the impact of back extensor strength on frailty outcomes outweighed the influence of age.

This study was not the first trial to use muscles other than hand grip muscles to reflect sarcopenia in frailty assessment. Previous studies have demonstrated associations between frailty and various muscle strengths and volumes, including the lower limb, forearm, and trunk [44,45]. However, studies that simultaneously investigate the relationship among muscle strength, muscle volume, and frailty are scarce. In this study, we evaluated both trunk muscle/fat volume and back extensor strength in the same group of participants. Through the concurrent analysis of muscle volume and muscle strength, our findings suggest a potential association between lower frailty status and increased back extensor strength, alongside increased volume in the abdominal and back muscles. In terms of rehabilitation medicine, exercise programs focused on strengthening back extensor strength by targeting the abdominal and back muscles may be beneficial for older adults.

Descriptive analysis revealed a difference in muscle/fat composition between sexes, even when considering both sexes had similar ages and BMIs. Specifically, males exhibited more visceral fat, whereas females had more subcutaneous fat. Previous studies explaining the hormonal differences between sexes supported these results. The primary male sex steroid, androgen, stimulated muscle growth and increased proliferation, contributing to higher levels of muscle mass in males [46]. Adipose tissues express estrogen receptors with higher activity in subcutaneous fat than in visceral fat. The elevated levels of estradiol in females mainly act through subcutaneous fat receptors, resulting in decreased lipolysis [47] and leading to higher SFM in females and higher VFM in males. 

The multivariate linear regression analysis, which examined the relationship between trunk muscle/fat composition and back extensor strength, showed that specific parts of the trunk were associated with muscle strength. These findings align with a previous study, which also reported a significant correlation between higher back extensor strength and increased body mass and non-fat body mass, as measured using the skinfold thickness evaluation from the Durnin and Womersley method [48]. In our study, we obtained trunk muscle volume measurements using CT scan. These specifically measured trunk muscle volumes revealed a significant linear relationship between increased back extensor strength and specific parts of the trunk, namely, the higher abdominal and back muscles.

This study showed that the XGBoost model had a low predictive power for Fried’s frailty, as measured by metrics such as AUC, accuracy, precision, recall, and f1 score. However, this outcome can be attributed to the multifactorial nature of Fried’s frailty criteria, including walking speed, physical activities, self-reported exhaustion, unintentional weight loss, and muscle strength. Using limited input variables such as age, sex, BMI, and back extensor strength resulted in the limited predictive power of this XGBoost model. Furthermore, we conducted the same multivariate logistic regression and XGBoost models using grip strength. The models using back extensor strength produced similar predictive results compared with those using grip strength, including ORs in the multivariate logistic regression analyses and feature importance in the XGBoost models. Additionally, the predictive power of back extensor strength, measured by the *p*-value in the multivariate logistic regression model and AUC, accuracy, precision, recall, and f1-score in the XGBoost model, was not inferior to that of grip strength. This implies that back extensor strength can be considered a useful alternative marker of frailty.

This study assessed trunk muscle strength owing to its substantial effect on health outcomes. Decreased muscle volume in the lower trunk region at the mid-lumbar level has been reported to be associated with pulmonary, hepatic, and systemic dysfunctions [18]. Furthermore, researchers have emphasized on back extensor strength because of its association with fall prevention in older adults, balance deficits, and other age-related symptoms. Older adults who have experienced falls tend to exhibit lower back extensor strength compared with those who have not [17]. Several studies showed that core strengthening programs, which targeted the maximal isometric strength of trunk flexors, extensors, and rotators, may delay frailty status by achieving improvements in various aspects such as spinal mobility (maximal extension to flexion and left to right flexion of the trunk), dynamic balance (stride velocity and the Functional Reach test), and functional mobility (Timed Up and Go test) [19,49].

There are several theories for using trunk muscle strength to assess frailty. The trunk serves as a kinetic link that facilitates the transfer of torque and angular momentum between the upper and lower extremities during various activities [20]. Therefore, core strength is a significant factor in everyday performance and sports-related activities for individuals of all ages [14]. Moreover, back extensor strength has been associated not only with the risk of dependence on activities of daily living and occupational skills in daily life [50] but also with multiple age-related symptoms, such as osteoporosis, low back pain, and Parkinson’s disease [51,52]. Based on these studies, we expected the significant correlations between back extensor strength and the aging process.

Our proposition of using back extensor strength as a potential marker of frailty aligns with the concept of a vicious cycle of frailty [53]. With aging, muscles experience changes in both quality and quantity, leading to an increase in interleukin-6 and C-reactive protein levels, which stimulate inflammatory mechanisms and the aging process [54]. These inflammatory changes can lead to chronic disorders associated with systemic low-level inflammation and decreased functionality in daily life [54,55]. Back extensor muscles contain more muscle cells than the forearm muscles that generate grip strength. Thus, by using back extensor muscles, we anticipated a more pronounced decrease in resting metabolism and physical activity, potentially exacerbating the aging process.

Previous studies explored the relationship between frailty and muscle strength in different body parts, such as the connection between lower-limb muscle strength and walking speed [56]. However, to the best of our knowledge, this is the first study to demonstrate that not only walking speed but also self-reported exhaustion was associated with muscle strength in different body parts, especially back extensor strength. This result suggests that back extensor strength can be considered a more general factor for frailty.

Our study does not propose that back extensor strength should replace grip strength as a measure of frailty. Instead, we propose back extensor strength as an additional option for evaluating frailty. Back extensor strength can be particularly valuable when grip strength measurements are impractical, such as in cases where a cast is present on the hand. Through the combined assessment of trunk muscle volume, we discovered the importance of trunk muscle volume in the aging process, suggesting that increasing trunk muscle strength may also be significant factor in preventing frailty. 

The clinical setting of this study can be considered as a useful approach for assessing frailty. Despite the widespread use of grip strength as a convenient measurement, its variability based on different postures, affecting the precision of the data, has been reported. Maximum grip strength is observed in a standing posture, with the shoulder fixed forward at 45 degrees, the elbow at 90 degrees, and the wrist and forearm in a neutral position [57]. Assessing back extensor strength was done in the sitting position, allowing for improved joint stabilization, especially in the hip and knee [27,58]. This controlled position ensures reliable measurements of back extensor strength, making it a potentially reliable indicator of muscle strength.

In the past, methods for measuring back extensor strength were expensive and cumbersome, acting as a barrier to data acquisition [59]. However, recent studies have used portable dynamometers, making back extensor strength measurements more applicable in clinical settings. Moreover, portable dynamometers provide data that were as accurate as those obtained using traditional methods [27]. As a result, we anticipate that the increased availability and accuracy of portable dynamometers may lead to more frequent analyses of back extensor strength in the future.

However, this study had two limitations. First, the participants consisted of farmers, who are likely to have higher levels of physical activity compared with the overall population. As a result, the findings may not fully represent the general population, which includes individuals with potentially lower physical activity levels and a higher likelihood of frailty. Second, frailty was defined using Fried’s criteria which focused on specific physical components. Adopting a broader definition of frailty that incorporates overall age-related biomarkers, such as hypertension, macular degeneration, and hearing loss, as the target outcomes of the prediction model could provide a clear understanding of the relationship between aging and muscle strength. By considering a more comprehensive definition of frailty, future studies may offer valuable insights into the multifaceted nature of frailty and its potential connections to muscle strength, facilitating more holistic approaches to frailty assessment and intervention in older adults.

## 5. Conclusions

This study assessed the potential of back extensor strength as an alternative marker of sarcopenia and frailty. Back extensor strength could serve as an alternative tool for evaluating frailty, and it is not inferior to grip strength. Our findings demonstrated a significant linear relationship between back extensor strength and the volumes of abdominal and back muscles. Furthermore, back extensor strength showed significant associations with multiple parameters of Fried’s frailty, making it a potential significant indicator of frailty. Therefore, exercise programs targeted at strengthening the abdominal and back muscles may be linked to a reduced frailty status. 

## Figures and Tables

**Table 1:** Participant characteristics by sex groups.

	Both (*n* = 560)	Male (*n* = 255)	Female (*n* = 305)	*p*-Value
Age	58.0 ± 7.0	58.5 ± 7.0	57.5 ± 6.9	0.130
Waist circumference (cm)	86.2 ± 9.3	89.6 ± 9.1	83.3 ± 8.5	<0.001
$\mathrm{BMI}\;(\mathrm{kg}/m^{2}$)	25.6 ± 3.1	25.6 ± 3.1	25.5 ± 3.0	0.989
TFM (cm^3^)	282.3 ± 93.6	272.7 ± 100.6	290.3 ± 86.7	0.059
VFM (cm^3^)	103.6 ± 45.5	116.1 ± 50.0	93.2 ± 38.4	<0.001
SFM (cm^3^)	178.7 ± 66.5	156.6 ± 63.1	197.1 ± 63.7	<0.001
TMM (cm^3^)	130.1 ± 30.0	155.9 ± 22.2	108.6 ± 15.2	<0.001
BMM (cm^3^)	57.6 ± 11.6	66.0 ± 9.3	50.6 ± 8.2	<0.001
PMM (cm^3^)	19.4 ± 6.9	25.3 ± 5.2	14.4 ± 3.2	<0.001
AMM (cm^3^)	53.2 ± 14.7	64.6 ± 12.9	43.7 ± 7.7	<0.001
Grip strength (Kgf)	28.7 ± 10.2	37.7 ± 7.2	21.3 ± 5.1	<0.001
Back extensor strength (N)	262.7 ± 93.8	321.0 ± 96.6	213.9 ± 55.9	<0.001
Walking speed (m/s)	1.0 ± 0.2	1.1 ± 0.2	1.0 ± 0.2	0.005
Unintentional weight loss (≥4.5 kg)	60 (10.7%)	24 (9.4%)	36 (11.8%)	0.660
Self-reported exhaustion (≥3 days/week)	44 (7.9%)	14 (5.5%)	30 (9.8%)	0.163
Physical activity (MET-min/week)	5622 ± 5657	5821 ± 5453	5455 ± 5827	0.673
Frailty score (%)				0.678
0	257 (45.9%)	113 (44.3%)	144 (47.2%)	
1	189 (33.8%)	97 (38.0%)	92 (30.2%)	
2	82 (14.6%)	35 (13.7%)	47 (15.4%)	
3	26 (4.6%)	8 (3.1%)	18 (5.9%)	
4	6 (1.1%)	2 (0.8%)	4 (1.3%)	

BMI, body mass index; TFM, total fat mass; VFM, visceral fat mass; SFM, superficial fat mass; TMM, total muscle mass; BMM, back muscle mass; PMM, psoas muscle mass; AMM, abdominal muscle mass. Values are expressed as mean ± standard deviation or *n* (%). *p* values are based on the t-test, chi-square test, and Fisher’s exact test.

**Table 2:** Participant characteristics by age groups.

	Age < 65 (*n* = 470)	Age ≥ 65 (*n* = 90)	*p*-Value
Sex (female %)	260 (55.3%)	45 (50%)	0.416
Waist circumference (cm)	85.9 ± 9.5	87.5 ± 8.2	0.099
$\mathrm{BMI}\;(\mathrm{kg}/m^{2}$)	25.6 ± 3.1	25.5 ± 2.8	0.758
TFM (cm^3^)	281.8 ± 93.4	284.6 ± 95.1	0.913
VFM (cm^3^)	102.4 ± 45.8	110.2 ± 43.5	0.051
SFM (cm^3^)	179.5 ± 66.0	174.4 ± 69.2	0.302
TMM (cm^3^)	131.7 ± 30.3	121.8 ± 27.3	0.008
BMM (cm^3^)	58.4 ± 11.4	53.1 ± 11.8	<0.001
PMM (cm^3^)	19.7 ± 7.0	17.6 ± 5.8	0.013
AMM (cm^3^)	53.6 ± 15.0	51.1 ± 13.1	0.219
Grip strength (Kgf)	29.2 ± 10.4	26.1 ± 8.9	0.020
Back extensor strength (N)	266.5 ± 93.4	242.4 ± 93.7	0.015
Walking speed (m/s)	1.1 ± 0.2	1.0 ± 0.1	<0.001
Unintentional weight loss (≥4.5 kg)	48 (10.2%)	12 (13.3%)	0.490
Self-reported exhaustion (≥3 days/week)	32 (6.8%)	12 (13.3%)	0.058
Physical activity (MET-min/week)	5646 ± 5573	5498 ± 6112	0.573
Frailty score (%)			<0.001
0	236 (50.2%)	21 (23.3%)	
1	157 (33.4%)	32 (35.6%)	
2	56 (11.9%)	26 (28.9%)	
3	16 (3.4%)	10 (11.1%)	
4	5 (1.1%)	1 (1.1%)	

BMI, body mass index; TFM, total fat mass; VFM, visceral fat mass; SFM, superficial fat mass; TMM, total muscle mass; BMM, back muscle mass; PMM, psoas muscle mass; AMM, abdominal muscle mass. Values are expressed as mean ± standard deviation or *n* (%). *p* values are based on the t-test, chi-square test, and Fisher’s exact test.

**Table 3:** Multivariate linear regression analysis for the predictor variable of back extensor strength.

	Coefficient	Standard Error	*t*	*p*-Value	VIF	Relative Weight
Constant	209.661	42.076	4.983	<0.001		
AMM	1.122	0.398	2.819	0.005	3.571	0.089
PMM	0.121	0.878	0.139	0.890	3.812	0.077
BMM	0.887	0.419	2.113	0.035	2.485	0.077
VFM	0.010	0.088	0.120	0.905	1.688	0.013
SFM	0.062	0.056	1.103	0.270	1.486	−0.005
Age	−1.823	0.508	−3.583	<0.001	1.312	−0.017
Sex	72.901	12.417	5.871	<0.001	3.985	−0.118

VIF, variance inflation factor; AMM, abdominal muscle mass; PMM, psoas muscle mass; BMM, back muscle mass; VFM, visceral fat mass; SFM, subcutaneous fat mass; Sex, female.

**Table 4:** Propensity score matching for back extensor strength and Fried’s frailty.

	Before Propensity Score Matching				After Propensity Score Matching			
	Low 20% Back Extensor Strength (*n* = 114)	Higher Back Extensor Strength (*n* = 444)	SMD	*p*-Value	Low 20% Back Extensor Strength (*n* = 108)	Higher Back Extensor Strength (*n* = 279)	SMD	*p*-Value
Age	60.6 ± 6.2	57.3 ± 7.0	0.532	<0.001	59.9 ± 5.7	59.4 ± 5.7	0.002	0.423
Female	54.4%	54.5%	−0.002	1.000	55.6%	55.9%	−0.019	1.000
Grip	25.4 ± 10.2	29.6 ± 10.0		<0.001	25.2 ± 10.3	28.7 ± 9.9		0.001
Wt. loss	13.2%	10.1%		0.447	13.0%	6.8%		0.082
Exhaustion	15.8%	5.6%		0.001	16.7%	6.8%		0.006
Activity	5372 ± 5051	5706 ± 5811		0.708	5340 ± 4998	5559 ± 5520		0.800
Gait speed	1.0 ± 0.2	1.1 ± 0.2		<0.001	1.0 ± 0.2	1.1 ± 0.2		0.002

SMD, standardized mean difference; grip, grip strength (N); wt. loss, unintentional weight loss (≥4.5 kg); exhaustion, self-reported exhaustion (≥3 days/week); activity, physical activity (MET-min/week); and gait speed (m/s).

**Table 5:** Result of the multivariate logistic regression analysis results for frailty in relation to back extensor strength.

Risk Factor	Coefficient	Standard Error	Odds Ratio (95% CI)	*p*-Value
Back Extensor strength	−0.009	0.003	0.990 (0.983–0.997)	0.008
BMI	0.026	0.061	1.027 (0.907–1.156)	0.664
Age	0.084	0.031	1.088 (1.025–1.160)	0.007
Sex	−0.108	0.488	0.897 (0.350–2.413)	0.824
Constant	−6.325			

CI, confidence interval; BMI, body mass index; Sex, female.

**Table 6:** Result of the multivariate logistic regression analysis results for frailty in relation to grip strength.

Risk Factor	Coefficient	Standard Error	Odds Ratio (95% CI)	*p*-Value
Grip strength	−0.140	0.037	0.869 (0.805–0.933)	<0.001
BMI	−0.002	0.061	0.997 (0.882–1.122)	0.967
Age	0.068	0.033	1.071 (1.005–1.145)	0.038
Sex	−1.320	0.650	0.267 (0.073–0.969)	0.042
Constant	−6.325			

CI, confidence interval; BMI, body mass index; Sex, female.

**Table 7:** Result of the XGBoost model for frailty prediction using back extensor strength.

Characteristics	Values
Feature importance in Fried’s frailty prediction	
Back extensor strength	0.502 ± 0.006
Age	0.325 ± 0.005
BMI	0.145 ± 0.005
Sex	0.026 ± 0.002
Predictive performance of XGBoost	
AUC	0.579 ± 0.004
Accuracy	0.71 ± 0.05
Precision	0.10 ± 0.01
Recall	0.56 ± 0.04
F1 score	0.15 ± 0.01

XGBoost, Extreme Gradient Boosting; BMI, body mass index; Sex, female; AUC, area under the curve.

**Table 8:** Result of the XGBoost model for frailty prediction using grip strength.

Characteristics	Values
Feature importance in Fried’s frailty prediction	
Grip strength	0.482 ± 0.007
Age	0.341 ± 0.006
BMI	0.153 ± 0.006
Sex	0.022 ± 0.002
Predictive performance of XGBoost	
AUC	0.676 ± 0.005
Accuracy	0.68 ± 0.02
Precision	0.09 ± 0.01
Recall	0.73 ± 0.02
F1 score	0.15 ± 0.01

XGBoost, Extreme Gradient Boosting; BMI, body mass index; Sex, female; AUC, area under the curve.

## Data Availability

The data underlying this study are available from the corresponding author upon reasonable request.
